# Factors associated with weaning success from prolonged mechanical ventilation in surviving Chinese pediatric intensive care units: a multicenter analysis

**DOI:** 10.3389/fped.2026.1737174

**Published:** 2026-02-20

**Authors:** Tian Li, Zhengzheng Zhang, Hong Ren, Chengjun Liu, Zihao Yang, Yibing Cheng, Wei Xu, Dong Qu, Hengmiao Gao, Furong Zhang, Hongjun Miao, Feng Ye, Musheng Li, Jianping Tao, Jianhui Zhang, Li Huang, Weiming Chen

**Affiliations:** 1 Department of Pediatric Intensive Care Unit, National Children’s Medical Center for South Central Region, Guangzhou Women and Children’s Medical Center, Affiliated Hospital of Guangzhou Medical University, Guangzhou, China; 2 Department of Pediatric Intensive Care Unit, National Children’s Medical Center, Children’s Hospital of Fudan University, Shanghai, China; 3 Department of Pediatric Intensive Care Unit, National Children’s Medical Center, Shanghai Children’s Medical Center, Shanghai Jiaotong University School of Medicine, Shanghai, China; 4 Department of Pediatric Intensive Care Unit, Western Pediatric Development Union, Children’s Hospital of Chongqing Medical University, Chongqing, China; 5 Department of Pediatric Intensive Care Unit, National Clinical Research Center for Child Health, Children’s Hospital of Zhejiang University School of Medicine, Hangzhou, Zhejiang, China; 6 Department of Pediatric Intensive Care Unit, Children’s Hospital Affiliated to Zhengzhou University, Zhengzhou, China; 7 Department of Pediatric Intensive Care Unit, National Children’s (Northeast) Regional Medical Center, Shengjing Hospital of China Medical University, Shenyang, China; 8 Department of Pediatric Intensive Care Unit, Childre’s Hospital, Capital Institute of Pediatrics, Beijing, China; 9 Department of Pediatric Intensive Care Unit, National Center for Children’s Health, Beijing Children’s Hospital, Capital Medical University, Beijing, China; 10 Department of Pediatric Intensive Care Unit, Wuhan Children’s Hospital, Tongji Medical College, Huazhong University of Science & Technology, Wuhan, Hubei, China; 11 Department of Emergency/Critical Medicine, Children’s Hospital of Nanjing Medical University, Nanjing, Jiangsu, China; 12 Department of Emergency, National Children’s Medical Centerr for South Central Region, Guangzhou Women and Children’s Medical Center, Affiliated Hospital of Guangzhou Medical University, Guangzhou, China

**Keywords:** children, difficult weaning, long-term ventilation, tracheostomy, ventilation strategies

## Abstract

**Objective:**

To describe factors of ventilation strategies associated with weaning success for **surviving** patients from prolonged mechanical ventilation (PMV) in Pediatric Intensive Care Units (PICUs).

**Methods:**

Conducted a retrospective study across eleven PICUs in mainland China from January 1, 2021, to December 31, 2022.

**Results:**

234 patients diagnosed with PMV were included in the study. Weaning Outcomes: 58.1% (136 patients) successfully weaned, includeing 11.1% (26 patients) required only a tracheostomy. 9.8% (23 patients) needed non-invasive ventilation. 32. 1% (75 patients) continued to require mechanical ventilation. 34.2% (80 patients) on invasive pressure control mode at PMV diagnosis. Pressure control was the most commonly used method. Synchronized intermittent mandatory ventilation (SIMV) used by 30.4% (71 patients). Pressure support ventilation (PSV) used by 5. 1% (12 patients). 63.2% (148 patients) received physiotherapy. 44.9% (105 patients) received cough augmentation techniques. 26.9% (63 patients) underwent tracheostomy after an average of 29 days of invasive mechanical ventilation. Higher fraction of inspired oxygen (FiO2) on PMV diagnosis day associated with weaning failure, the OR value is 0.674. While lower airway diseases had one more times chance of weaning seccess than central nervous system diseases, the OR value is 2.144.

**Conclusion:**

Among survivors, ventilation strategies for PMV weaning in Chinese PICUs are diverse, with pressure control commonly used initially, followed by SIMV and PSV. We identified a higher FiO2 at PMV diagnosis as risk factors for weaning failure, while lower airway diseases were easier to wean.

## Introduction

With advancements in medical technology, an increasing number of patients are surviving in the pediatric intensive care unit (PICU). However, some of these patients may not fully recover and may require life support equipment, such as mechanical ventilators, for extended periods. This presents a significant challenge for critical care providers and families worldwide(1–3). In developed Western countries, children who are dependent on technology often receive care from home care nurses and social workers, along with support from the community and government. In contrast, this model of child life support has not been widely implemented in mainland China.

In China, critically ill children who are still on ventilators but in relatively stable condition are typically transferred to national or regional children's medical centers for further treatment. However, none of these PICUs permit family members to accompany their children, and there are no alternative units available to care for these ventilator-dependent patients. The PICU is the only facility where families can remain with their children. Some families may opt to discontinue treatment due to financial constraints. However, an increasing number of families are choosing home ventilation to facilitate their children's reintegration into family life and society.

However, there is limited data available regarding patients requiring prolonged mechanical ventilation (PMV) nationwide, and follow-up studies are scarce. In this context, our multicenter collaborative group was established to further investigate medical management strategies and outcomes for children with PMV. This includes examining factors such as mechanical ventilation modes and settings, physiotherapy, tracheostomy, and other interventions, with the aim of improving outcomes for this population.

## Materials and methods

### Study design

This study was a multicenter retrospective cohort study, initially approved by the Institutional Review Board of the National Children's Medical Center at Fudan University [No.(2020)475]. For retrospective analysis of desensitized data, the study received an exemption from informed consent from the IRB. The study protocol was registered at Clinicaltrial.gov NCT04511741.

Eleven tertiary PICUs of eight major cities in mainland China participated in the survey: Beijing, Shanghai, Guangzhou, Chongqing, Hangzhou, Zhengzhou, Wuhan, and Nanjing. Among these, two were designated as national children's medical centers, while four were classified as regional children's medical centers.

This study specifically focused on children who survived to the weaning assessment phase. All centers had followed standardized protocols, including weaning(4), sedation(5), airway clearance techniques(6) and nutrition(7).

### Definitions

PMV was defined according to the *National Association of Medical Direction of Respiratory Care (NAMDRC)* definition(8), which was 21 consecutive days of ventilation for more than 6 h per day considering invasive and noninvasive ventilation duration and including short interruptions (<48 h) of ventilation during the weaning process as the same episode of ventilation.

Ventilator associated pneumonia (VAP) was defined (9) as a new and persistent(>48 h) or a progressive radiographic infiltrate plus two of the following: temperature of >38℃ or <36℃, blood leukocyte count of >10,000 cells/ml or <5,000 cells/ml, purulent tracheal secretions, and gas exchange degradation.

Spontaneous breathing trial (SBT) was conducted using a T-piece, continuous positive airway pressure (CPAP), or pressure support (PS) for a duration of at least 30 min and no longer than 120 min. The criteria for a successful SBT was respiratory rate, heart rate, and blood pressure within the normal range or less than a 20% change from baseline, saturation >90% on FiO2 < 40%, no signs of increased work of breathing.

Successful weaning (10) was difined as liberation from mechanical ventilation for >48 consecutive hours. A patient was classified as weaned regardless of whether the tracheostomy tube was subsequently decannulated or remained in place. The presence of a tracheostomy alone did not constitute weaning failure.

### Data collection

From January 1, 2021, to December 31, 2022, we retrospectively included all patients with PMV who were admitted to PICUs. Inclusion criteria: (1)aged between 28 days and 18 years; (2) met the definition of PMV. The exclusion criteria were: (1) patients who died during their PICU stay; (2) patients who had their treatment withdrawn for any reason. One clinician at each unit was trained to collect information and enter it into an online database.

The information collected included: (1) demographic information such as, age, gender, and weight; (2) the admitting diagnosis and the causes of ventilator dependence, which included central nervous system diseases, neuromuscular disorders, upper airway diseases, lower airway diseases, cardiovascular diseases, and others; (3) the ventilator mode and settings on the day that met the definition of PMV, as well as at 14, 28, and 60 days thereafter until PICU discharge; (4) the occurrence of VAP during the PICU stay; (5) the time to tracheostomy; and (6) adjunctive therapies, including sedation (continuous infusion of any type of sedative or pain control medication), paralysis, vasoactive drug infusion (including epinephrine, norepinephrine, dopamine, dobutamine, and milrinone), physiotherapy (including passive movements, active exercises, and extracorporeal diaphragmatic electrical stimulation), and airway clearance techniques (such as hypertonic saline, postural drainage and percussion, and cough augmentation techniques).

### Statistical analysis

All statistical analyses were conducted using SPSS version 24.0 (SPSS, Chicago, IL). Descriptive statistics were summarized for the entire study population as well as for subgroups. Patient characteristics were presented as counts and percentages, while continuous variables that were not normally distributed were summarized using the median and interquartile range (IQR). The Chi-squared test was employed for categorical variables. The Mann–Whitney U test or Kruskal–Wallis rank test was utilized for two-sample or multi-sample continuous data that did not follow a normal distribution. The primary outcome was a binary variable: successful weaning at dischargevs. still ventilator-dependent at discharge. Logistics regression analyses were performed to evaluate the relationships between variables and outcomes. The hazard ratio and confidence interval were reported for each variable. A *p*-value of less than 0.05 was considered statistically significant for all analyzed parameters. A Cox proportional hazards regression was performed to examine the robustness of our findings, with time zero defined as the first day of meeting prolonged mechanical ventilation criteria. Patients who were discharged while still ventilator-dependent were censored at the date of discharge.

## Results

### Characteristics and outcomes of PMV patients

**Figure 1:**
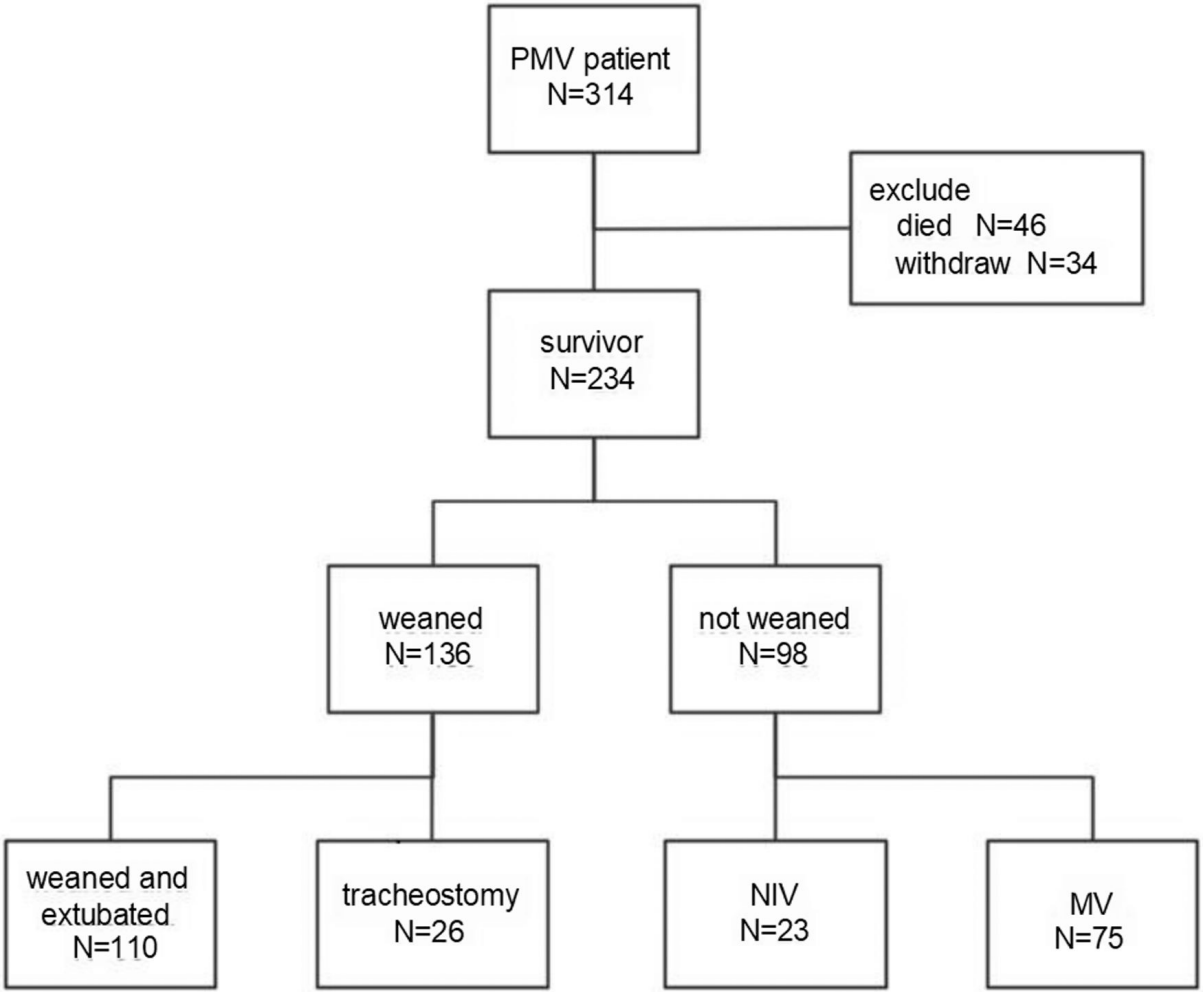
Flowchart of PMV patients.

A total of 314 patients met the PMV diagnosis during the study period. Of these, 46 patients died, and 34 patients withdrew from the PICU (see Figure 1). By the time of discharge from the PICU, 136 patients (58. 1%) had been successfully weaned from invasive mechanical ventilation. This group included 110 patients who required no respiratory support and 26 patients who needed only tracheostomy. However, 98 patients (41.9%) still required mechanical ventilation. Among these, 23 patients were decannulated and needed non-invasive ventilation, while 75 patients were transferred to a recovery center, community hospital, hospice unit, or home, remaining on full-time invasive mechanical ventilation.

**Figure 2:**
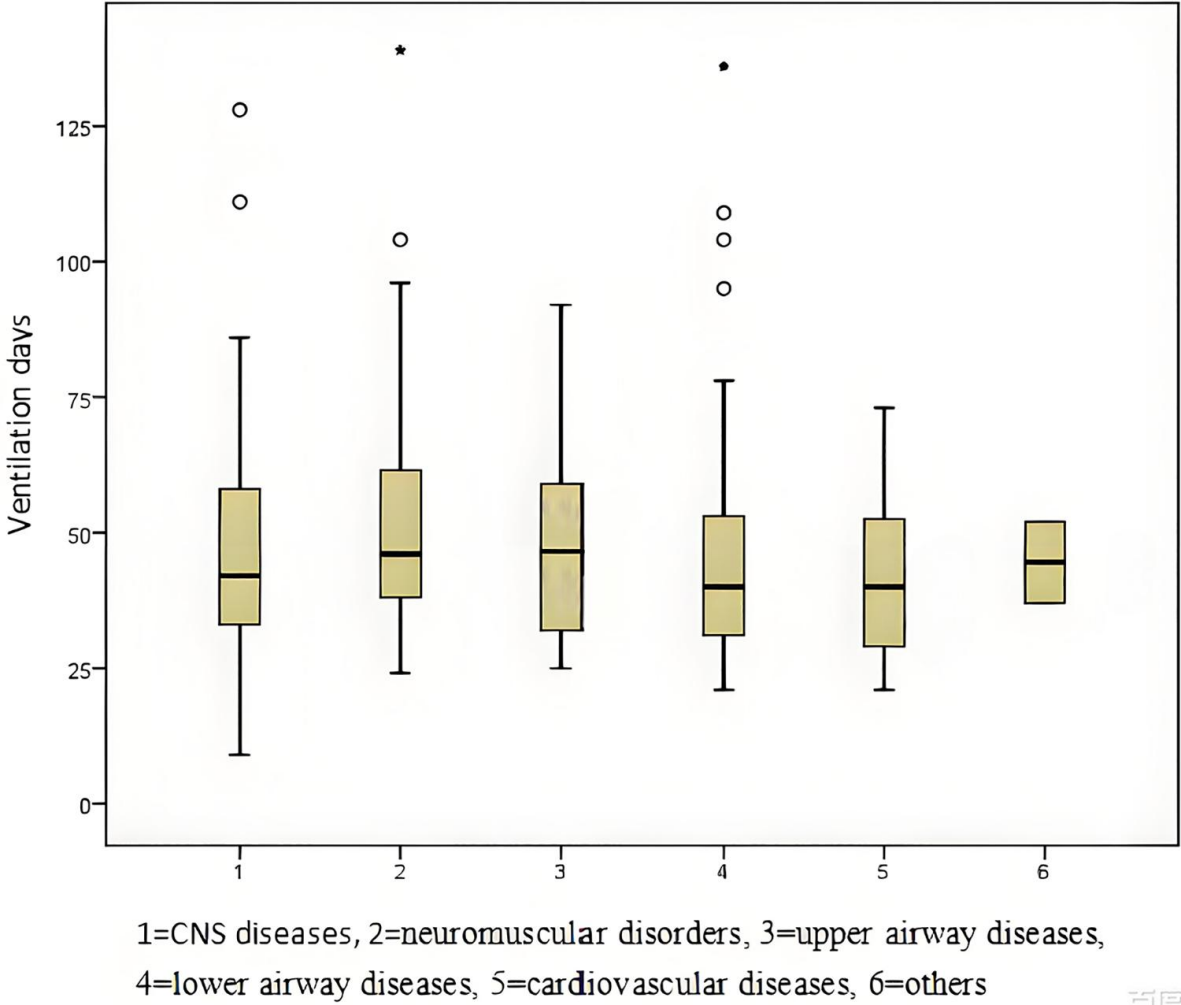
Mean duration of ventilation according to the causes of ventilator dependence.

The characteristics and data of PMV patients are presented in Table 1. The average age of PMV patients was 31.5 months, with 56.4% being male. Central nervous system diseases (97 patients, 41.5%) were the primary cause of ventilator dependence, followed by lower airway diseases (73 patients, 31.2%) and neuromuscular disorders (37 patients, 15.8%). The average duration of ventilation is illustrated in Figure 2.

**Table 1:** Characteristics of PMV patients.

Characteristic	Total *N* = 234	Weaned *N* = 136	Not weaned *N* = 98	*P*
Age, month, median (IQR)	31.5 (7–84.75)	28 (6–81.75)	40.4 (13–95.75)	0.054
Weight, kg, median (IQR)	12.0 (7.0–22.625)	11.25 (6.125–21.0)	13.75 (8.15–24)	0.095
Gender, male, *n* (%)	132 (56.4)	69 (50.7)	63 (64.3)	0.039
Causes of ventilator dependence, *n* (%)	<0.001			
central nervous system diseases	97 (41.5)	53 (39.0)	44 (44.9)	
neuromuscular disorders	37 (15.8)	11 (8.1)	26 (26.5)	
upper airway diseases	14 (6.0)	11 (8.1)	3 (3.1)	
lower airway diseases	73 (31.2)	54 (39.7)	19 (19.4)	
cardiovascular diseases	11 (4.7)	6 (4.4)	5 (5.1)	
others	2 (0.9)	1 (0.7)	1 (1.0)	
Before the day of PMV diagnosis				
SBT, *n* (%)	81 (34.6)	46 (33.8)	35 (35.7)	0.764
Extubation, *n* (%)	77 (32.9)	51 (37.5)	26 (26.5)	0.078
Reintubation, *n* (%)	60 (77.9)	39 (76.5)	21 (80.8)	0.210
On the day of PMV diagnosis				
PELOD-2	5 (4–7)	5 (4–7)	4.5 (3–7)	0.461
Mechanical ventilation mode, *n* (%)	0.575			
Invasive-control	110 (47.0)	60 (44.1)	50 (51.0)	
Invasive-support	100 (42.7)	61 (44.9)	39 (39.8)	
NIV	24 (10.3)	15 (11.0)	9 (9.2)	
Mechanical ventilation settings, median (IQR)				
FiO2, %	35 (30–40)	35 (30–40)	40 (30–45)	0.022
PIP, cmH2O	16 (12–20)	16 (10–20)	17 (14–21)	0.153
PEEP, cmH2O	5 (4–6)	5 (4–6)	5 (4–5)	0.515
Sedation, *n* (%)	143 (61.1)	97 (71.3)	46 (46.9)	<0.001
Vasoactive drug infusion, *n* (%)	27 (11.5)	13 (9.6)	14 (14.3)	0.264
VAP, *n* (%)	75 (32.1)	46 (33.8)	29 (29.6)	0.494
Physiotherapy, *n* (%)	148 (63.2)	84 (61.8)	64 (65.3)	0.579
Airway clearance techniques, *n* (%)	215 (91.9)	124 (91.2)	91 (92.9)	0.642
Cough assist techniques, *n* (%)	105 (44.9)	60 (44.1)	45 (45.9)	0.785
Tracheostomy, *n* (%)	63 (26.9)	26 (19.1)	37 (37.8)	0.002
Time to tracheostomy, d, median (IQR)	29 (16–39)	28 (17.25–38)	29 (15.5–41.5)	0.743
PICU stay, d, median (IQR)	41.5 (31–60.25)	40.5(31.25–58)	43.5(31–63)	0.335

IQR, interquartile range; PMV, prolonged mechanical ventilation; SBT, spontaneous breathing trail; PELOD-2, pediatric logistic organ dysfunction-2; NIV, non-invasive ventilation; PIP, peak inspiratory pressure; PEEP, positive end-expiratory pressure; VAP, ventilator associated pneumonia.

### Ventilation strategies for PMV patients

Before the day of PMV diagnosis, the most common ventilation mode was pressure control (162 patients, 69.2%). Fifteen patients (6.4%) received high-frequency oscillatory ventilation (HFOV), and two patients received neurally adjusted ventilatory assist (NAVA). Only one patient did not receive invasive mechanical ventilation. Two units preferred synchronized intermittent mandatory ventilation (SIMV) as the initial ventilation mode, while the other nine units favored control modes, particularly pressure control.

There were a total of 81 patients (34.6%) who underwent a SBT prior to the diagnosis of PMV, and 36 patients (44.4%) underwent the trial more than once. On average, 1 SBT (range: 1–2) was performed per patient. A total of 77 patients attempted extubation, among children who ultimately progressed to PMV, 60 of these patients (77.9%) had failed an initial extubation attempt prior to PMV diagnosis and required reintubation within 48 h. Additionally, there were 5 patients who extubated without undergoing an SBT.

On the day of PMV diagnosis, 110 patients (47.0%) were still on the invasive-control mode of ventilation, with pressure control being the most commonly used mode (80 patients, 34.2%). SIMV and PSV were the most popular strategies employed to shorten ventilation, accounting for 71 patients (30.4%) and 12 patients (5. 1%), respectively. Additionally, 24 patients (10.3%) were receiving non-invasive ventilation. Ventilation settings showed no significant differences between the weaning success and failure groups, except for FiO2, which was significantly higher in the not weaned group [40% [30%–35%] vs. 35% [30%–40%], *P* = 0.022]. Furthermore, 61. 1% of patients were using sedative drugs, with a significantly higher percentage in the weaning success group compared to the not weaned group. Additionally, 11.5% of patients were on at least one vasoactive drug infusion.

Overall, 63.2% of patients with PMV received physiotherapy, which included passive limb movements (136/148), active limb exercises (5/148), muscle electrical stimulation (26/148), and respiratory muscle training (25/148). Additionally, 91.9% of patients underwent airway clearance techniques, including hypertonic saline (107/215), postural drainage and percussion (174/215), and cough assist techniques (105/215). The incidence of VAP during the stay in the PICU was 32. 1%.

### Tracheostomy

A total of 63 patients underwent tracheostomy (Table 2). The median age of these patients was 60 months (range: 25–114 months), which was significantly older than the median age of those who did not receive a tracheostomy, which was 25 months (range: 5–77 months), with a *p*-value of <0.001. Among the patients who received tracheostomy, 55 (87.3%) had central nervous system (CNS) or neuromuscular diseases. The median time from the initiation of mechanical ventilation to the tracheostomy procedure was 29 days (range: 16–39 days). In comparison to patients who did not undergo tracheostomy, those who did required less sedation (42.9% vs. 67.8%, *p* = 0.001) and fewer vasoactive drugs (3.2% vs. 14.6%, *p* = 0.015). Additionally, they received more physiotherapy (84. 1% vs. 55.6%, *p* < 0.001) and had a longer length of stay in the PICU [median of 54 days [range: 40–81 days] vs. 39 days [range: 29–53 days], *p* < 0.001].

**Table 2:** Characteristics of patients with tracheostomy.

Characteristic	Total *N* = 234	Tracheostomy *N* = 63	Without tracheostomy *N* = 171	*P*
Age, month, median (IQR)	31.5 (7–84.75)	60 (25–114)	25 (5–77)	<0.001
Age group, month, *n* (%)				<0.001
<12 m	74 (31.6)	7 (11.1)	67 (39.2)	
≥12 m, <36 m	49 (20.9)	17 (27.0)	32 (18.7)	
≥36 m, <72 m	38 (16.2)	11 (17.5)	27 (15.8)	
≥72 m,	73 (31.2)	28 (44.4)	45 (26.3)	
Weight, kg, median (IQR)	12.0 (7.0–22.625)	16.4 (10.0–29.0)	11.0 (6.0–18.0)	<0.001
Gender, male, *n* (%)	132 (56.4)	39 (61.9)	93 (54.4)	0.304
Causes of ventilator dependence, *n* (%)				<0.001
Central nervous system diseases	97 (41.5)	38 (60.3)	59 (34.5)	
Neuromuscular disorders	37 (15.8)	17 (27.0)	20 (11.7)	
Upper airway diseases	14 (6.0)	4 (6.3)	10 (5.8)	
Lower airway diseases	73 (31.2)	4 (6.3)	69 (40.4)	
Cardiovascular diseases	11 (4.7)	0 (0)	11 (6.4)	
Others	2 (0.9)	0 (0)	2 (1.2)	
Before the day of PMV diagnosis				
SBT, *n* (%)	81 (34.6)	26 (41.3)	55 (32.2)	0.194
Extubation, *n* (%)	77 (32.9)	19 (30.2)	58 (33.9)	0.587
Reintubation, *n* (%)	60 (77.9)	19 (100)	41 (70.7)	0.008
On the day of PMV diagnosis				
PELOD-2	5 (4–7)	4 (3–7)	5 (4–7)	0.109
Mechanical ventilation mode, *n* (%)				0.005
Invasive-control	110 (47.0)	30 (47.6)	80 (46.8)	
Invasive-support	100 (42.7)	33 (52.4)	67 (39.2)	
NIV	24 (10.3)	0 (0)	24 (14.0)	
Mechanical ventilation settings, median (IQR)				
FiO_2_, %	35 (30–40)	35 (30–40)	35 (30–45)	0.324
PIP, cmH_2_O	16 (12–20)	15 (10.25–17.75)	18 (13.25–22)	0.008
PEEP, cmH_2_O	5 (4–6)	5 (4–5)	5 (4–6)	0.006
Sedation, *n* (%)	143 (61.1)	27 (42.9)	116 (67.8)	0.001
Vasoactive drug infusion, *n* (%)	27 (11.5)	2 (3.2)	25 (14.6)	0.015
VAP, *n* (%)	75 (32.1)	20 (31.7)	55 (32.2)	0.952
Physiotherapy, *n* (%)	148 (63.2)	53 (84.1)	95 (55.6)	<0.001
Airway clearance techniques, *n* (%)	215 (91.9)	59 (93.7)	156 (91.2)	0.547
Cough assist techniques, *n* (%)	105 (44.9)	28 (44.4)	77 (45.0)	0.936
Weaned, *n* (%)	136 (58.1)	26 (41.3)	110 (64.3)	0.002
PICU stay, d, median (IQR)	41.5 (31–60.25)	54(40–81)	39(29–53)	<0.001

We further divided the tracheostomy patients into two groups based on the timing of their tracheostomy (Table 3). Patients who received a tracheostomy within three weeks had a shorter stay in the PICU.

**Table 3:** Tracheostomy timing.

Characteristic	Total *N* = 63	≤3 weeks *N* = 24	>3 weeks *N* = 39	*P*
Age, month, median (IQR)	60 (25–114)	85.5 (31.25–112.25)	43 (24–119)	0.358
Age group, month, *n* (%)				0.265
<12m	7 (11.1)	1 (4.2)	6 (15.4)	
≥12 m, <36 m	17 (27.0)	6 (25.0)	11 (27.0)	
≥36 m, <72 m	11 (17.5)	3 (12.5)	8 (20.5)	
≥72 m,	28 (44.4)	14 (58.3)	14 (35.9)	
Weight, kg, median (IQR)	16.4 (10–29)	21.25 (9.625–37)	15 (10–28)	0.488
Gender, male, *n* (%)	39 (61.9)	14 (58.3)	25 (64.1)	0.647
Causes of ventilator dependence, *n* (%)				0.607
Central nervous system diseases	38 (60.3)	17 (70.8)	21 (53.8)	
Neuromuscular disorders	17 (27.0)	5 (20.8)	12 (30.8)	
Upper airway diseases	4 (630)	1 (4.2)	3 (7.7)	
Lower airway diseases	4 (630)	1 (4.2)	3 (7.7)	
VAP, *n* (%)	20 (31.7)	7 (29.2)	13 (33.3)	0.730
Weaned, *n* (%)	26 (41.3)	9 (37.5)	17 (43.6)	0.634
PICU stay, d, median (IQR)	54 (40–81)	39(32.25–62)	59(45–87)	0.002

In the logistics regression analyses (Table 4), the causes of ventilator dependence was associated with weaning, lower airway diseases experienced easier weaning compared to central nervous system diseases (OR = 2.144, 95% CI: 1.251–3.672). And every 10% absolute increase in FiO₂, the risk of weaning failure increased by approximately 33% (OR = 0.674, 95% CI: 0.542–0.837). The findings were robust in a sensitivity analysis using a Cox proportional hazards model, which yielded fully consistent results (data not shown).

**Table 4:** Factors associated with weaning success in children with PMV.

Factor	OR (95% confidence interval)	*p*
Gender		
Male	1(ref)	
Female	1.898 (1.273–2.829)	0.002
Causes of ventilator dependence		
Central nervous system diseases	1(ref)	
Neuromuscular disorders	0.607 (0.291–1.264)	0.182
Upper airway diseases	1.855 (0.894–3.848)	0.097
Lower airway diseases	2.144 (1.251–3.672)	0.006
Cardiovascular diseases	1.857 (0.758–4.548)	0.176
Others	1.855 (0.236–14.569)	0.557
FiO_2_ on the day of PMV diagnosis (per 10%)	0.674 (0.542–0.837)	<0.001
Sedation on the day of PMV diagnosis	1.125 (0.712–1.776)	0.613

Adjust for diagnostic category, centers, severity, ventilation modes/settings on the day of PMV diagnosis.

## Discussion

This is a retrospective study examining ventilation strategies for weaning patients with PMV across multiple national and regional children's medical centers in mainland China, utilizing a representative sample of cases. The definition of PMV in children varies(11); however, we have chosen to use the NAMDRC definition for comparison. Among the patients with PMV, 14.6% (46 out of 314) died in the PICU, which is comparable to the 15.9% mortality rate reported in China from 2017 to 2019 (12), but significantly lower than the 29% reported in a previous review(2). This lower mortality rate may be attributed to the fact that these ventilator-dependent patients received continuous intensive care in the PICU.

We included only survivors in our study of ventilation strategies for weaning. Excluded population (died/withdraw)'s characteristics was showed in Supplementary Table S1. Our findings indicated that 58. 1% (136 out of 234) of the patients were successfully weaned from mechanical ventilation prior to their discharge from the PICU. This result is comparable to the 50% weaning rate observed before discharge from the hospital in a meta-analysis of adult patients(13).

Among all study populations, the highest proportion of cases requiring PMV was attributed to central nervous system (CNS) diseases (41.5%), followed by lower airway diseases (31.2%). In European and American countries(14, 15), the proportion of delayed withdrawal of treatment due to neuromuscular diseases and congenital metabolic disorders was the highest. However, in China, due to unique social and medical conditions, a significant number of patients with congenital diseases opted for immediate withdrawal of treatment following diagnosis.

Compared to China's data from 2017 to 2019 (12), the proportion of PMV attributed to lower airway diseases exhibited a downward trend, decreasing from 41.6% to 31.2%. This decline may be attributed to several factors. First, the COVID-19 pandemic prompted the implementation of stringent national control policies, including mandatory mask-wearing, which contributed to a reduction in the overall incidence of respiratory infectious diseases. Second, advancements in the diagnosis and treatment of respiratory diseases, along with an improved understanding of mechanical ventilation and programmed weaning strategies, have facilitated earlier extubation for many children suffering from lower airway diseases. Additionally, many children's hospitals have established separate cardiac intensive care units (CICUs), which were not included in our study; consequently, the proportion of cardiovascular diseases accounted for only 4.7%.

We surveyed the ventilation strategies employed for patients with PMV and compared our findings to those from several years ago (12). We discovered that all patients received invasive mechanical ventilation, except for one patient who only received non-invasive ventilation. Most units preferred control modes, particularly pressure control, while only two of the eleven units opted for SIMV mode as the initial choice. By the 21st day of ventilation, 47% of patients remained on control mode. When considering weaning from mechanical ventilation, the strategies employed included SIMV, PSV, and CPAP. Prior to extubation, only 34.6% of patients underwent a SBT. This low percentage may be attributed to the high prevalence of central nervous system and neuromuscular diseases among the patients. Non-invasive ventilation was frequently utilized to prevent or manage post-extubation respiratory failure in high-risk patients. Due to the retrospective nature of this study and the lack of standardized weaning protocols across different units, we were unable to determine which approach was superior to the others. NAVA has been implemented in recent years, and research in adults has shown that NAVA improves diaphragm efficiency, unlike PSV (16). However, only two patients in our study received NAVA. The advantages and disadvantages of NAVA, as well as its potential to shorten ventilation time, still require further investigation in pediatric patients.

Early mobilization and physiotherapy(17) are crucial adjunctive therapies that play significant roles in successful weaning. The ATS/CHEST guidelines recommend initiating mobilization as early as 24 h after ventilation in adults (18). During ventilation, 63.2% of patients received physiotherapy, with the majority undergoing passive limb movements; only five patients participated in active exercises. Additionally, the case report form we designed did not capture the initial timing and frequency of mobilization, which hindered our ability to identify differences between the weaning success or not. However, in the tracheostomy group, the proportion of patients receiving physiotherapy was considerably higher, likely due to 87.3% of these patients having central nervous system and neuromuscular diseases. In the absence of physiotherapists, these patients were more likely to receive physiotherapy.

Since a strong cough can predict extubation success and may reduce ICU length of stay and mortality (19), cough augmentation techniques are employed to facilitate extubation (20). However, the results indicate that these techniques seem to have no effect on weaning.

In our study, tracheostomy was performed on 26.9% of the patients requiring PMV, which is comparable to Cinotti's report (21) of 25.6%, although they defined PMV as lasting more than 10 days. Half of the tracheostomies (32 out of 63) were conducted in two PICUs in Shanghai. In China, parental decision-making regarding long-term ventilation is influenced by various factors, including national healthcare policies, economic conditions, uncertain prognoses, and psychological stress. These factors complicate the decision-making process for parents (22). In Shanghai, an international metropolis, both healthcare providers and family members demonstrate a higher acceptance of tracheostomy compared to other regions in China. Additionally, Shanghai benefits from relatively higher economic levels, better medical insurance, and stronger community support, contributing to the increased proportion of tracheostomy procedures performed.

The average time to perform a tracheostomy was 29 days after the initiation of mechanical ventilation, significantly longer than the 10–12 days reported for adults (23, 24) and also longer than our data from 2017 to 2019, which indicated an average of 21 days (12). In pediatric patients, this duration is considered a late tracheostomy, regardless of the definitions of early tracheostomy, which are defined as occurring before 7 days of intubation according to Holscher et al. (25) or within 14 days as per Lee et al. (26) Both of these studies suggested that early tracheostomy reduces the use of sedatives and shortens the length of stay in the PICU, although they found no impact on the incidence of hospital-acquired pneumonia. In our study, all tracheostomies were classified as late, and the length of stay in the PICU was significantly longer for the tracheostomy group. However, among tracheostomy patients, those who underwent the procedure within three weeks had a shorter PICU stay compared to those who had late tracheostomies. This discrepancy may be partly attributed to epidemic prevention and control policies that limited the flow of patients, as well as the absence of specialized units for stable patients. Families of these patients spent considerable time in the PICU to receive training in necessary knowledge and skills before returning home or to rehabilitation centers.

Our study suggests that the causes of ventilator dependence is associated with weaning outcomes. Central nervous system (CNS) diseases were identified as the most common cause of PMV. In comparison to this subgroup, the chance of weaning success in patients with lower airway diseases had one more times. It is understandable that CNS diseases require a longer recovery time. On the day of PMV diagnosis, each 10% increase in FiO_2_ was associated with a 33% increase in the risk of weaning failure.

As a retrospective study, our data collection from case records is inadequate. Nevertheless, this study can represent the current status of children on PMV in mainland China. There are still unresolved questions that need to be addressed through well-designed multicenter clinical randomized controlled trials (RCTs) examining various protocols for weaning strategies, physiotherapy, tracheostomy, and other related interventions. Specialized weaning centers should be established by governments and healthcare providers. Our study population was restricted to children who survived to undergo weaning assessment. By excluding non-survivors (a group with inherently higher illness severity), our identified predictors of weaning success are specifically applicable to this survivor cohort and should not be generalized to all children who develop prolonged mechanical ventilation. Our assessment of sedation and analgesia was limited to a binary classification (“any use”). We lacked granular data on medication type (e.g., analgesic vs. sedative), dosage, depth of sedation, or specific treatment targets. This heterogeneity limits precise interpretation of its association with weaning outcomes.

## Conclusions

In our multicenter retrospective study, the characteristics of pediatric patients requiring PMV who survived in the PICU showed minimal variation. The most common cause of ventilator dependence was central nervous system diseases. The proportion of successful weaning in the ICU was low, and the timing of extubation and tracheostomy varied significantly. Among survivors, patients with lower airway diseases are easier to wean, while a higher FiO2 on the day of PMV diagnosis was associated with not weaned by the time of PICU discharge.

## Data Availability

The original contributions presented in the study are included in the article/Supplementary Material, further inquiries can be directed to the corresponding authors.
