# Supplementary material for: Factors associated with weaning success from prolonged mechanical ventilation in surviving Chinese pediatric intensive care units: a multicenter analysis
Source: Front Pediatr. 2026 Feb 20;14:1737174. doi: 10.3389/fped.2026.1737174 (PMC12963309; doi:10.3389/fped.2026.1737174)
Supplement: Supplementary file 1 [file Table1.docx]

Supplemental material Table 1. Sensitivity analysis of died/withdrew

| Characteristics | Total  N=314 | Included  N=234 | Excluded  (died/withdrew)  N=80 | P |
| --- | --- | --- | --- | --- |
| Gender, male, n (%) | 181(57.6) | 132(56.4) | 49(61.3) | 0.449 |
| Causes of ventilator dependence, n (%) | | | | 0.113 |
| central nervous system diseases | 124(39.5) | 97(41.5) | 27(33.8) |  |
| neuromuscular disorders | 44(14.0) | 37(15.8) | 7(8.8) |  |
| upper airway diseases | 18(5.7) | 14(6.0) | 4(5.0) |  |
| lower airway diseases | 106(33.8) | 73(31.2) | 33(41.3) |  |
| cardiovascular diseases | 17(5.4) | 11(4.7) | 6(7.5) |  |
| others | 5(1.6) | 2(0.9) | 3(3.8) |  |
| On the day of PMV diagnosis |  |  |  |  |
| PELOD-2 | 5(4-7) | 5(4-7) | 7(5-9) | <0.001 |
| Mechanical ventilation mode, n (%) | | | | 0.003 |
| Invasive-control | 163(51.9) | 110(47.0) | 53(66.3) |  |
| Invasive-support | 126(40.1) | 100(42.7) | 26(32.5) |  |
| NIV | 25(8.0) | 24(10.3) | 1(1.3) |  |
| Mechanical ventilation settings, median (IQR) | | | | |
| FiO_2_, % | 35(30-45) | 35(30-40) | 40(31.5-52.5) | 0.021 |
| PIP, cmH_2_O | 18(13-22) | 16(12-20) | 20(15-22) | 0.009 |
| PEEP, cmH_2_O | 5(4-6) | 5(4-6) | 6(5-7) | 0.001 |
| Sedation, n(%) | 195(62.1) | 143(61.1) | 52(65.0) | 0.536 |
| Vasoactive drug infusion, n(%) | 53(16.9) | 27(11.5) | 26(32.5) | <0.001 |
| Tracheostomy, n (%) | 71(22.6) | 63(26.9) | 8(10.0) | 0.002 |
